# Stimulus-Timing Dependent Multisensory Plasticity in the Guinea Pig Dorsal Cochlear Nucleus

**DOI:** 10.1371/journal.pone.0059828

**Published:** 2013-03-20

**Authors:** Seth D. Koehler, Susan E. Shore

**Affiliations:** 1 Kresge Hearing Research Institute, Department of Otolaryngology, University of Michigan Medical School, Ann Arbor, Michigan, United States of America; 2 Department of Molecular and Integrative Physiology, University of Michigan Medical School Ann Arbor, Michigan, United States of America; 3 Department of Biomedical Engineering, University of Michigan, Ann Arbor, Michigan, United States of America; University of Salamanca- Institute for Neuroscience of Castille and Leon and Medical School, Spain

## Abstract

Multisensory neurons in the dorsal cochlear nucleus (DCN) show long-lasting enhancement or suppression of sound-evoked responses when stimulated with combined somatosensory-auditory stimulation. By varying the intervals between sound and somatosensory stimuli we show for the first time *in vivo* that DCN bimodal responses are influenced by stimulus-timing dependent plasticity. The timing rules and time courses of the observed stimulus-timing dependent plasticity closely mimic those of spike-timing dependent plasticity that have been demonstrated *in vitro* at parallel-fiber synapses onto DCN principal cells. Furthermore, the degree of inhibition in a neuron influences whether that neuron has Hebbian or anti-Hebbian timing rules. As demonstrated in other cerebellar-like circuits, anti-Hebbian timing rules reflect adaptive filtering, which in the DCN would result in suppression of sound-evoked responses that are predicted by activation of somatosensory inputs, leading to the suppression of body-generated signals such as self-vocalization.

## Introduction

Fusiform cells in the dorsal cochlear nucleus (DCN) integrate auditory and somatosensory information [1], [2], [3]. Responses to sound in these multisensory neurons, the principal output neurons of the DCN, remain enhanced or suppressed for up to two hours following bimodal somatosensory and auditory stimulation [4]. The mechanisms underlying this long-lasting effect have not been elucidated, but the duration of the effect is consistent with synaptic plasticity.

**Figure 1:**
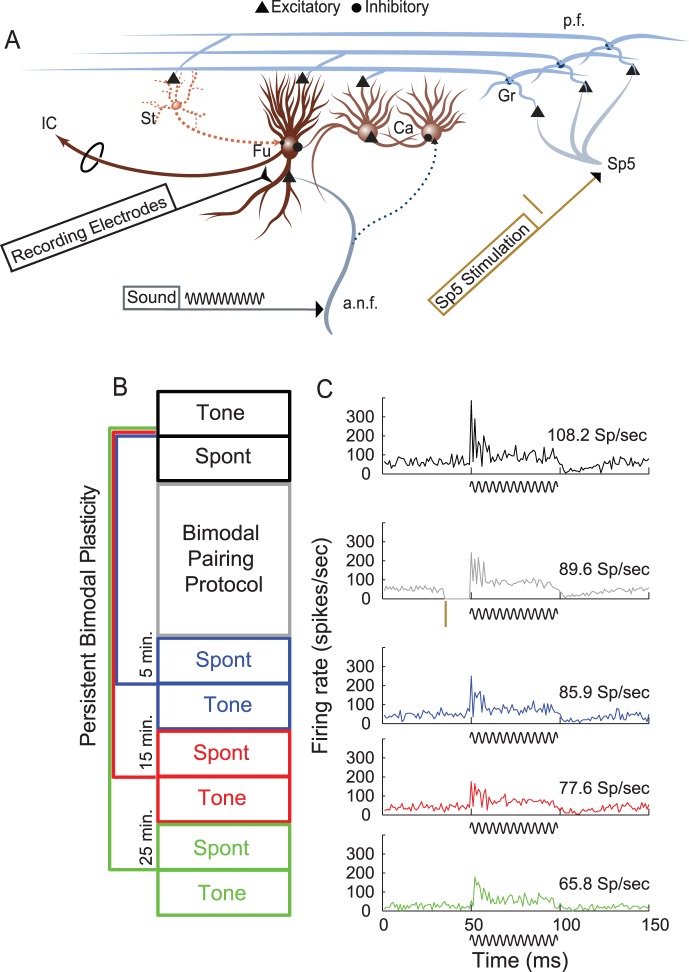
Bimodal plasticity recorded *in vivo* from DCN. A. Schematic of stimulation and recording locations in Sp5 and DCN. Thirty two-channel recording electrodes (black) spanned all layers across the tonotopic axis of the DCN. Short current pulses delivered via a bipolar stimulating electrode (brown) placed into Sp5 activated parallel fiber inputs to DCN. Tones were delivered through calibrated, hollow ear bars. B. The bimodal plasticity recording protocol consisted of tones presented immediately before (black), 5 minutes after (blue), 15 minutes after (red), and 25 minutes after (green) the bimodal pairing protocol (gray). C. Post stimulus time histograms and mean firing rate over the duration of the 50 ms tone stimulus showing responses to sound in one DCN unit before (black), during (grey), 5 minutes after (blue), 15 minutes after (red) and 25 minutes after (green) bimodal stimulation. The stimulus cartoons below each PSTH demonstrate the tone (black sinusoid) presented alone or preceded by electrical pulses in Sp5 (brown tick). Due to artifact contamination, spikes immediately following Sp5 stimulation were removed (second histogram from top). Bin width = 1 ms. Ca - cartwheel cell; Fu - fusiform cell; Gr - granule cell; St – Stellate cell; IC - inferior colliculus; Sp5 - spinal trigeminal nucleus; a.n.f - auditory nerve fiber; p.f - parallel fiber.

Specialized spike-timing dependent plasticity (STDP) has been demonstrated *in vitro* at parallel fiber synapses with DCN neurons [5] and might underlie *in vivo* long-lasting bimodal plasticity [4]. Parallel-fiber axons from cochlear nucleus granule cells, which receive somatosensory inputs [6], [7], synapse on the apical dendrites of both fusiform cells and their inhibitory interneurons, cartwheel cells (Fig. 1A). Parallel fiber synapses on fusiform and cartwheel cells exhibit Hebbian and anti-Hebbian STDP, respectively, which is induced by the close temporal association of fusiform cell action potentials with excitatory post-synaptic potentials elicited by pre-synaptic action potentials in parallel fibers [5], [8]. Hebbian STDP is induced when synaptic activity preceding a post-synaptic spike potentiates the synapse while synaptic activity following a post-synaptic spike depresses the synapse. In contrast, anti-Hebbian STDP is induced when synaptic activity preceding a post-synaptic spike depresses the synapse while synaptic activity following a post-synaptic spike potentiates the synapse [9].

DCN models [10] and studies of cerebellar-like structures in electrosensory fish [11], [12] suggest that STDP may be a generalized learning mechanism for adaptive filtering in early sensory processing centers. DCN neural responses may adapt over time to emphasize or de-emphasize features of auditory signal representation that are temporally associated with non-auditory afferent [1], [13] or top-down feedback [14], [15] signals supplied through the granule cell network. In this study, we supplied sub-threshold synaptic activity to DCN neurons via the granule cell network by stimulating spinal trigeminal nucleus (Sp5) neurons. The excitatory terminals of these somatosensory neurons, which process vocal feedback signals and other facial somatosenations, end in the granule cell domain [6], [7], [16]. We have previously shown that pairing somatosensory stimuli with sound has a long-lasting influence on DCN responses to sound [4]. Here, we examined STDP as an underlying mechanism for multisensory plasticity by varying the relative timing and order of the auditory (equivalent to post-synaptic) and somatosensory (equivalent to pre-synaptic) components of bimodal stimuli following standard stimulus-timing dependent protocols [17]. Our results show for the first time *in vivo* that long-lasting bimodal plasticity in the DCN is stimulus-timing dependent and thus likely to be driven by STDP at parallel fiber synapses.

## Results

Bimodal plasticity induction in the DCN was assessed *in vivo* by measuring sound-evoked and spontaneous firing rates before and after bimodal stimulation. Bimodal stimulation consisted of electrical pulses delivered to Sp5 to activate parallel fiber-fusiform and -cartwheel cell synapses, paired with a 50-ms tone burst to elicit spiking activity in fusiform and cartwheel cells (Fig. 1A). Dorsal cochlear nucleus unit responses to unimodal tones and spontaneous activity following bimodal stimulation were recorded with a multi-channel electrode placed into the DCN using a standard protocol (Fig. 1B). Bimodal stimulation could either suppress or enhance responses to sound. In the representative unit shown in Figure 1C, spontaneous activity and responses to tones were suppressed 5, 15, and 25 minutes after bimodal stimulation. Bimodal enhancement and suppression in this study refer to unimodal (auditory) response magnitudes at different times after bimodal stimulation compared to unimodal response magnitudes before bimodal stimulation. These are equivalent to the “late” or long-lasting changes previously described [4]that reflect plasticity. This “bimodal plasticity” contrasts with bimodal integration in which bimodal enhancement and suppression were measured by comparing responses during bimodal stimulation with unimodal (auditory) responses [1], [3], [18].

### Bimodal Plasticity is Stimulus-timing Dependent

**Figure 2:**
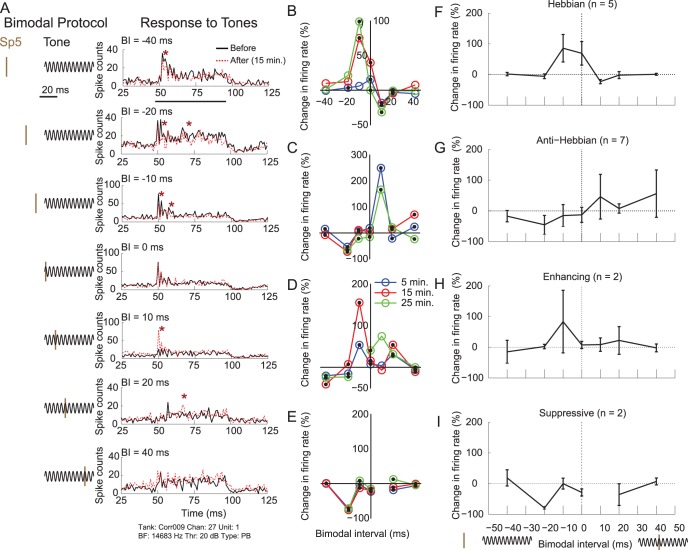
Bimodal plasticity in DCN is stimulus-timing dependent. A. PSTHs of sound-evoked responses for one single unit 15 minutes after (red dashed line) bimodal stimulation differ from PSTHs of responses to the same sound before (black solid line) bimodal stimulation. The stimulus cartoons to the left of each PSTH identify the bimodal interval between the electrical pulses in Sp5 (brown) and the tone (black) for each bimodal pairing protocol. The bimodal interval (BI) was randomly varied from somatosensory (Sp5) preceding sound by 40 ms (top) to somatosensory following the onset of sound by 40 ms (bottom). Stars highlight regions of enhancement or suppression. The horizontal bar below the top PSTH indicates the sound stimulus duration. B. Hebbian-like stimulus-timing dependence shown in one DCN single unit. C. Anti-Hebbian-like stimulus-timing dependence shown in one DCN single unit. D. Enhancement-only stimulus-timing dependence shown in a DCN single unit. E. Suppression-only stimulus-timing dependence shown in a DCN single unit. B–E. Blue, red, and green lines indicate the change in firing rate 5, 15, and 25 minutes after bimodal stimulation, respectively. F–I. Mean single-unit timing rules at 15 minutes after bimodal stimulation are grouped by timing rule: Hebbian, anti-Hebbian, enhancing, and suppressing units are shown from top to bottom. Number of single units shown in parentheses above each panel. Error bars represent mean +/−95% confidence interval.

*In vivo* stimulus timing dependent plasticity has been shown to reflect underlying Hebbian and anti-Hebbian STDP [17]. To assess stimulus-timing dependence in the present study, the bimodal stimulation protocol (Fig. 1B) was repeated with varying bimodal intervals: i.e., Sp5 stimulation onset minus sound onset. In a representative unit (Fig. 2A) the auditory response was suppressed after bimodal stimulation when somatosensory (Sp5) preceded auditory stimulation but was enhanced if auditory preceded somatosensory stimulation. Negative bimodal intervals indicate somatosensory preceding auditory while positive bimodal intervals indicate auditory preceding somatosensory stimulation. Bimodal plasticity was considered stimulus-timing dependent when the sound-evoked firing rates increased or decreased following bimodal stimulation at some, but not all, of the bimodal intervals tested. All units in which responses to sound were modulated by the bimodal pairing protocol showed stimulus-timing dependence (i.e. the firing rate increased or decreased by at least 20% following at least one bimodal interval tested; 16/16 single-unit and 98/110 multi–unit clusters).

For each unit demonstrating stimulus-timing-dependent plasticity, a timing rule was constructed from the percent change in firing rate as a function of bimodal interval (Fig. 2B–E). Timing rules were classified into Hebbian-like (Fig. 2B), anti-Hebbian-like (Fig. 2C), enhanced (Fig. 2D), or suppressed (Fig. 2E). Mean single unit timing rules for each group are shown in Figures 2F–2I. Hebbian-like units were maximally enhanced when somatosensory preceded auditory stimulation and maximally suppressed when auditory preceded somatosensory stimulation, likely reflecting Hebbian STDP at the parallel-fusiform cell synapse (n = 5; Fig. 2B, 2F). Anti-Hebbian-like units were maximally suppressed when somatosensory preceded auditory stimulation and maximally enhanced when auditory preceded somatosensory stimulation (n = 7; Fig. 2C, 2G). Other units were either enhanced (n = 2; Figs. 2D, 2H) or suppressed (n = 2; Figs. 2E, 2I) by all bimodal pairing protocols. Comparison of single and multi unit clusters indicated that the same Hebbian-like (n = 25), anti-Hebbian-like (n = 18), enhanced (n = 18), suppressed (n = 12) timing rules were observed in multi-unit clusters. Thirty three multi units showed a complex dependence of suppression and enhancement on the bimodal interval (not shown).

### Bimodal Enhancement and Suppression Stabilize after 15 Minutes and Begins to Recover by 30 Minutes

**Figure 3:**
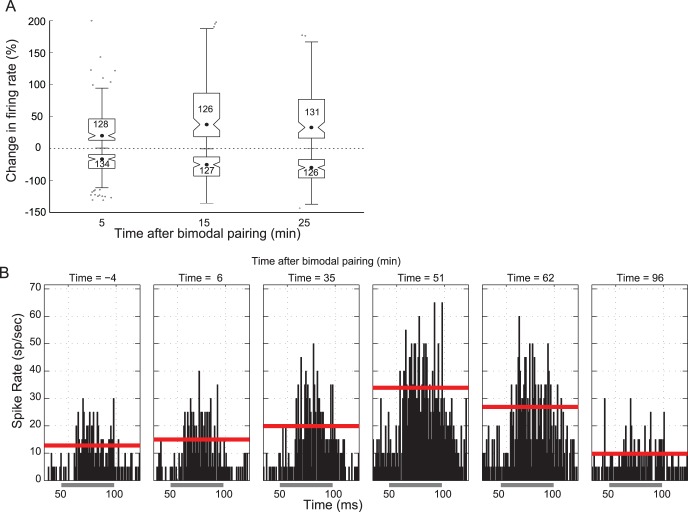
Maximal plasticity continues to develop for 15 minutes and begins to recover by 30 minutes. A. The median and interquartile range for enhancement (top half) and suppression (bottom half). Only the maximum bimodal-induced enhancement and suppression are included. Inward indentations on the bars indicate 95% confidence intervals. Each box is labeled with the number of units. B. PSTHs of responses from an example unit showing maximal bimodal plasticity at 50 minutes post-pairing followed by recovery at 91 minutes post pairing. The time in minutes relative to the bimodal pairing trials is listed above each panel. The solid red line indicates the mean firing rate measured over the duration of the tone (50–100 ms). Gray bar below the x-axis indicates the duration of the tone stimulus.

Synaptic plasticity at parallel fiber synapses in the DCN develops over the course of several minutes [5]. To compare the bimodal plasticity time course to synaptic plasticity time courses, bimodal plasticity was measured 5, 15, and 25 minutes after bimodal stimulation (Fig. 1B) for both single and multi units. The maximal enhancement and suppression and the bimodal interval that induced maximal enhancement and suppression were used to estimate the effect of bimodal stimulation on the DCN neural population. The change in firing rate following bimodal stimulation was often greater at 15 or 25 minutes than at 5 minutes after bimodal stimulation (Fig. 2B–E). Maximal bimodal enhancement plateaued 15 minutes following bimodal pairing and started to recover at 25 minutes (Fig. 3A top). In contrast, maximal bimodal suppression continued to develop over 25 minutes (Fig. 3A bottom). Median maximal suppression was −28% (n = 126) after 25 minutes while median maximal enhancement was 40% (n = 126) by 25 minutes after bimodal pairing. These data indicate that tone responses began to recover towards baseline 25 minutes after bimodal pairing. In some units, responses to tones recovered to baseline levels within 90 minutes after the bimodal pairing (Fig. 3B).

### The DCN Neural Population is Dominated by Anti-Hebbian-like Stimulus-timing Dependent Plasticity

**Figure 4:**
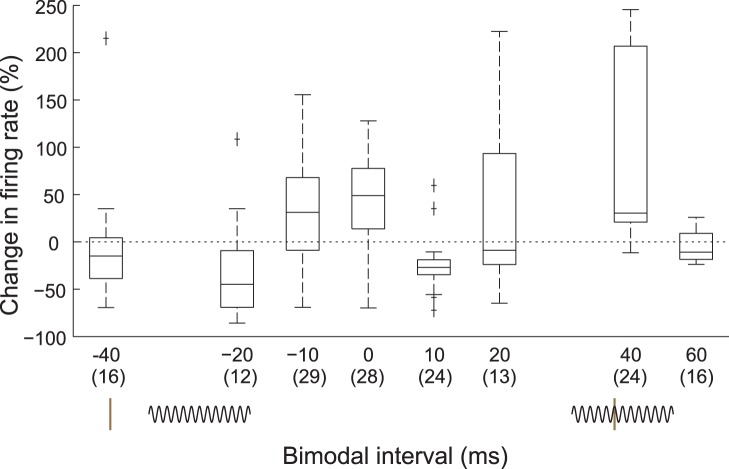
Distribution of preferred bimodal intervals. At 15 minutes, bimodal intervals of 0 and 40 ms induce maximal enhancement while bimodal intervals of −20 and +10 ms induce maximal suppression. Box and whisker plots indicate median and interquartile ranges of the maximum change in sound-evoked firing rates. Number of units shown in parentheses below the x-axis.

Identifying the maximum bimodal enhancement and suppression with corresponding bimodal intervals allowed a population estimate of the stimulus-timing dependence of bimodal plasticity in the DCN (Fig. 4). When the most effective bimodal pairing protocol consisted of Sp5 following tone stimulation by 20 or 40 ms, Sp5 synchronous with tone stimulation, or Sp5 preceding tone stimulation by 10 ms, bimodal stimulation was most likely to produce enhancement. In contrast, when the most effective bimodal stimulation protocol was Sp5 preceding tone stimulation by 20 or 40 ms or following tones by 10 ms, it primarily induced bimodal suppression.

### Bimodal Stimulation Induced Stronger Persistent Effects than Unimodal Stimulation

**Figure 5:**
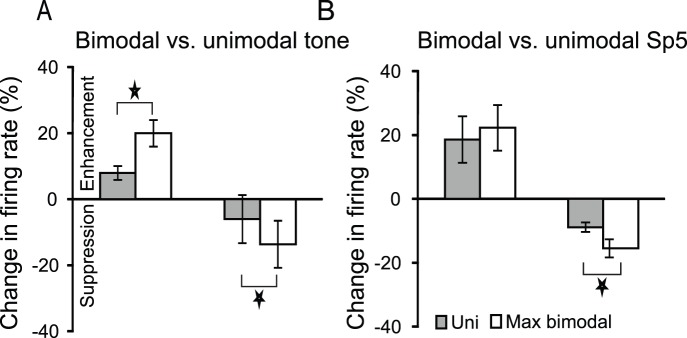
Bimodal stimulation has a greater long-lasting effect than unimodal stimulation. A. Unimodal sound stimulation (grey bars) induced significantly less enhancement and suppression than maximally effective bimodal stimulation (white bars). B. Unimodal somatosensory stimulation (grey bars) induced significantly less suppression, but not significantly less enhancement, than maximally effective bimodal stimulation (white bars). Sp5– spinal trigeminal nucleus. Stars indicate significance by paired t-test (p<0.05).

Our proposed hypothesis that STDP underlies long-lasting bimodal plasticity requires that paired auditory and somatosensory stimulation induce long-lasting suppression or enhancement of tone-evoked responses. To test this, changes in unimodal tone-evoked responses were measured during protocols in which the bimodal stimulus was replaced by a unimodal stimulus (either sound or Sp5 stimulation alone). Maximal bimodal enhancement (1-tailed paired Student’s t-test; n = 10; p = 0.025) and suppression (1-tailed paired Student’s t-test; n = 17; p = 0.00023) were significantly stronger than enhancement or suppression of the tone-evoked response following unimodal tone stimulation (Fig. 5A). However, only maximal suppression (1-tailed paired Student’s t-test; n = 20; p = 0.017), but not enhancement (1-tailed paired Student’s t-test; n = 7; p = 0.24), following bimodal stimulation was stronger than that following unimodal Sp5 stimulation (Fig. 5B). Thus, activation of both somatosensory and auditory inputs have a greater long-lasting affect on DCN unit responses than either activation of auditory or somatosensory inputs aone.

### Units Excited by Sp5 Stimulation Exhibited Hebbian Timing Rules while Units Inhibited by Sp5 Stimulation Exhibited Anti-Hebbian Timing Rules

**Figure 6:**
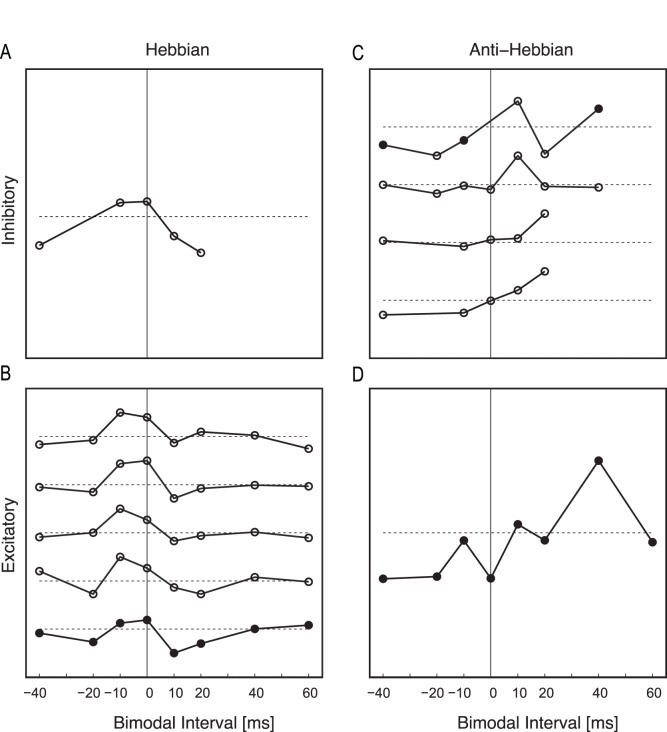
Bimodal timing rules depend on the unimodal somatosensory response. Each plot contains the timing rule from one or more unit, normalized to the maximum change, shifted vertically and centered on the horizontal dashed lines. Empty circles represent mult-unit activity while filled circuits represent single-unit activity. A–B. Units with Hebbian like timing rules with A. inhibitory or B. excitatory responses to Sp5 stimulation. C–D. Units with anti-Hebbian-like timing rules with C. inhibitory or D. excitatory response to Sp5 stimulation.

Activation of somatosensory neurons has previously been shown to elicit excitation, inhibition, or complex responses in DCN neurons [1], [13]. Somatosensory stimulation elicits either excitatory or inhibitory responses in a particular fusiform cell depending on whether input is conveyed to that fusiform cell directly from parallel fiber inputs or via inhibitory interneurons (cartwheel cells). Although Sp5 stimulation amplitude was selected to activate subthreshold somatosensory inputs, eleven units had measurable excitatory or inhibitory responses to unimodal Sp5 stimulation and clearly defined Hebbian or anti-Hebbian timing rules. Five out of 6 units that responded to Sp5 stimulation with excitatory responses exhibited Hebbian timing rules, suggesting that Hebbian timing rules were driven by parallel fiber-to-fusiform cell synapses (Fig. 6A and 6B). In contrast, four out of 5 units that responded to Sp5 stimulation with inhibition exhibited anti-Hebbian timing rules, suggesting anti-Hebbian dependence on parallel fiber-to-cartwheel cell synapses (Fig. 6C and 6D). Units that did not show clear stimulus-timing dependency were just as likely to be excited or inhibited by Sp5 stimulation alone (not shown).

### Stimulus Timing Rules Correlate with Inhibitory Inputs

**Figure 7:**
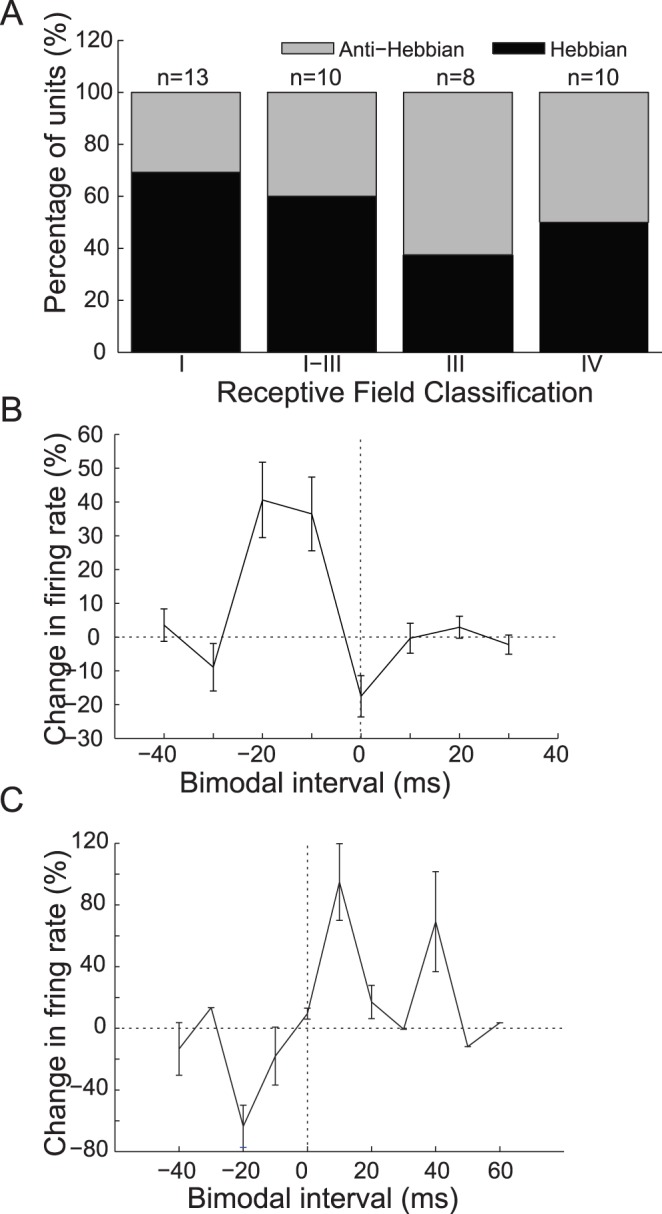
Sound-driven timing rules across unit response types. A. The proportion of Hebbian-like (black) and anti-Hebbian-like (gray) timing rules observed in units with principal (fusiform or giant) cellresponse areas. The number of units included in each group is shown at the top of each bar. B. Mean timing rules from 16 units with either B or PB and Type I or II physiological classifications. All 16 units had Hebbian-like timing rules. C. Mean timing rules from 4 units with onset and Type IV or IV-T physiological classifications. All 4 units had anti-Hebbian-like timing rules. B–C. Error bars indicate +/− S.E.M.

Units were classified according to traditional physiological response schemes for guinea pig [19] by their frequency response maps (n = 63 units; types I, II, III, I–III, IV, and IV-T) and their temporal responses properties at best frequency (n = 66 units; buildup, pause-buildup, chopper, onset, and primary-like). These physiological response properties are linked to intrinsic, morphological, and network properties of DCN neurons, including their somatosensory innervation. In the present study, the proportion of units with Hebbian and anti-Hebbian-like timing rules correlated with the degree of inhibition reflected in their response areas. Figure 7A shows the proportion of Hebbian and anti-Hebbian-like units for types I, I–III, III, and IV, response map classifications usually associated with fusiform or giant cells [20]. Hebbian-like timing rules were more likely to be found in units with Type I response areas with no inhibition than in units with Type III or IV response areas with significant inhibition away from best frequency or at high intensities.

Timing rules and the strength of bimodal plasticity were also compared for groups of units with each combination of temporal and receptive field response types. Two classes of neurons had consistent bimodal timing rules. Buildup or pauser-buildup units with type I or type II response areas exhibited clear Hebbian-like timing rules (Fig. 7B). In contrast, onset units with type IV or IV-T response maps exhibited only anti-Hebbian timing rules (Fig. 7C).

### Spontaneous Rate Changes Correlate with Changes in Sound-evoked Firing Rate

**Figure 8:**
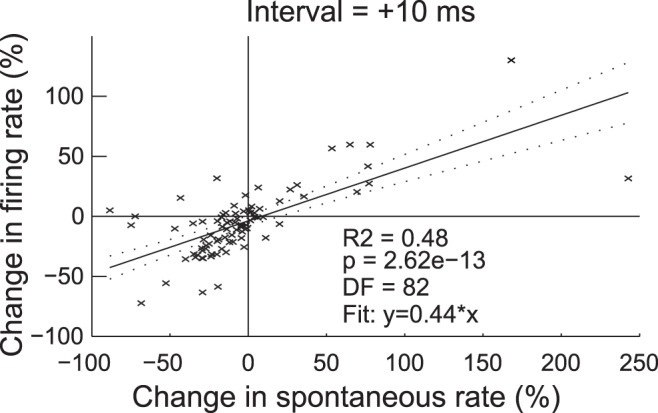
Spontaneous activity timing rules across unit response types. Linear regression analysis of the change in firing rate and the change in spontaneous rate fifteen minutes following bimodal stimulation with a bimodal interval of +10 ms. The solid line represents the best fit model with parameters designated on the figure. Dashed lines represent confidence intervals.

After bimodal stimulation the changes in sound-driven and spontaneous firing rates were significantly correlated for all bimodal intervals except for −20 ms (0.21< R^2^<0.48). The highest correlation in sound-driven and spontaneous firing rates was observed following the +10 ms bimodal interval (Fig. 8A, linear regression analysis, DF = 82; R^2^ = 0.48; p = 2.62e^−13^). However, changes in sound-evoked and spontaneous firing rates were not significantly correlated following bimodal stimulation at an interval of −20 ms.

## Discussion

### Evidence for STDP-driven Bimodal Plasticity in DCN

Bimodal stimulation of auditory and somatosensory inputs to the DCN modulates spontaneous and sound-driven activity in a manner consistent with STDP at parallel fiber synapses with fusiform cells. This stimulus-timing dependent bimodal plasticity in the DCN exhibits timing rules that reflect those found *in vitro* at parallel fiber-fusiform and parallel fiber-cartwheel cell synapses [5]. The time course of bimodal enhancement, which plateaus 15 minutes post-pairing, and bimodal suppression, which continues to develop 25 minutes post-pairing, are also consistent with the time course of STDP. *In vitro* STDP recordings at parallel fiber synapses revealed maximum LTP 2-to-20 minutes after STDP induction and maximum LTD 5-to-15 minutes after STDP induction [8]. Stimulus-timing dependence in the auditory [21] and visual [22] cortices develops over 3–5 minutes while STDP in the electrosensory lobe of the mormyrid takes 5–10 minutes to develop [11]. Adaptive filtering theories suggest that, after plasticity induction, responses should recover to baseline with repeated unimodal tone stimulation [23]. Enhanced, but not suppressed, firing rates in the present study began to recover towards baseline within 30 minutes but only a few units (Fig. 3B) showed complete recovery within 90 minutes of bimodal stimulation. This recovery duration is consistent with STDP given the duration of both potentiating and depressing DCN STDP observed *in vitro* [5], [8]. The kinetics and requirements for recovery from bimodal plasticity deserve careful study to identify whether responses recover spontaneously to baseline with time or whether sound stimulation is necessary for recovery.

The equivalent enhancement in DCN induced by bimodal stimulation and Sp5 stimulation alone (Fig. 5B) suggests that synaptic or intrinsic mechanisms, independent of STDP, are partially involved in the long-lasting somatosensory influence on fusiform cell responses to sound. At parallel fiber synapses in the DCN, high frequency synaptic stimulation also induces LTP while low frequency synaptic stimulation induces LTD [24]. In addition, spiking activity in cartwheel cells induces retrograde endocannabinoid release, which suppresses parallel fiber input to cartwheel cells and could potentially enhance the fusiform cell sound-driven response through release from cartwheel cell inhibition [25]. Parallel fiber [26], [27] and bimodal stimulation [2], [3] have also been shown to induce changes in intrinsic firing properties of fusiform cells through de-inactivation of K^+^ channels, although these mechanisms have only been shown to be effective on the timescale of seconds.

If STDP at parallel fiber synapses is the underlying mechanism for bimodal plasticity, then there must be a mechanism by which synaptic plasticity at parallel fiber synapses on fusiform cell apical dendrites influences fusiform cell responses to auditory nerve input on their basal dendrites. One possibility, heterosynaptic plasticity, is unlikely given that STDP at parallel fiber synapses on fusiform cells is homosynaptic and does not affect remote synapses on either apical or basal dendrites [5], [24]. A more likely possibility is that synaptic plasticity at parallel fiber synapses broadens the window for temporal summation in fusiform cells by shifting the resting membrane potential, leading to a decrease in the spike generation threshold at auditory nerve synapses, as demonstrated by modeling and in vitro experiments in fusiform cells [28]. Stimulus-timing dependent plasticity in other cell types in the DCN, such as giant cells or vertical cells, which receive few or no parallel fiber inputs may depend in a similar manner on as yet undescribed STDP mechanisms. Fusiform cell stimulus-timing dependent properties may also be conveyed to other DCN cell types via axon collaterals [29]. Although the targets of these collaterals have not been well-described, fusiform cell axon collaterals have been shown to synapse on giant cell dendrites [30] and correlation analysis suggests the existence of excitatory intra-DCN connectivity in guinea pigs [31].

### Network and Intrinsic Properties Influence Bimodal Plasticity

The timing rule continuum, from Hebbian-like to anti-Hebbian-like to complex, shown in the present study is not surprising given the variety of DCN neural types and suggests that intrinsic or network mechanisms act alongside STDP to control bimodal plasticity. Bimodal plasticity timing rules *in vivo* may also be influenced by cholinergic input from the superior olivary complex or the tegmental nuclei [32], [33], [34], which modulate STDP in the DCN, converting Hebbian LTP to anti-Hebbian LTD at parallel fiber-fusiform cell synapses [35].

The finding that physiological classes of DCN neurons exhibit differing stimulus-timing dependencies implies that physiological (and likely morphological) subtypes of DCN neurons perform different functions with their multimodal inputs. The present data indicate that DCN neurons with less inhibitory influence (Type I receptive fields) are more likely to display Hebbian-like stimulus timing dependence while those with significant inhibitory influence (Type III and IV receptive fields) are more likely to display anti-Hebbian-like stimulus timing dependence. This may reflect inhibitory influences from vertical cell or cartwheel cells on post-synaptic spiking patterns which, in fusiform cells, are likely determined by long-lasting or pre-hyperpolarizing inhibition [36]. The timing rules for STDP induction in other systems depend not only on the relative timing of pre-synaptic activity and post-synaptic spikes, but also on the number and pattern of post-synaptic spikes [37].

Alternatively, the source of sound-driven inhibition to DCN principal cells may also exhibit predominantly Hebbian-like stimulus-timing dependent plasticity, resulting in anti-Hebbian-like timing rules in recipient neurons. One source could be type II neurons, putative vertical cells. Type II neurons supply inhibition to fusiform and giant cells [38], are inhibited by somatosensory and parallel fiber input [13], and in our data exhibit Hebbian-like stimulus-timing dependent plasticity. Future studies should thus consider the functional connectivity of non-auditory inputs via granule or other cells to different classes of principal cells and how they might shape the spectral selectivity of DCN neurons.

### The Role of STDP in Adaptive Processing

Hebbian and anti-Hebbian STDP are important mechanisms for adaptive processing in cerebellar-like circuits. Neural responses to predictable stimuli in these circuits exhibit long-lasting adaptation induced by correlations between primary sensory input and error signals supplied by motor control or secondary sensory inputs [9], [39], [40]. The present study describes the first *in vivo* experiments evaluating mechanisms for multisensory adaptive processing in the DCN. Adaptive processing in the DCN has been proposed as a mechanism to suppress responses to sound predicted by non-auditory signals [10], [41], such as self-generated sound preceded by somatosensory input [1], [42]. It also may adapt sound localization signals in the DCN [43] to pinna or head position [3], [42], [44]. A high proportion of DCN neurons exhibited anti-Hebbian-like timing rules, with responses to tones suppressed when Sp5 stimulation preceded the tone and enhanced when the tone preceded Sp5 stimulation. This observation is consistent with the hypothesis that DCN neurons cancel self-generated sounds predicted by preceding somatosensory activation. Future studies addressing adaptive processing should use natural stimuli that would likely activate a smaller group of fibers with less synchronous input to the DCN.

### Implications for Tinnitus

Reports of elevated spontaneous firing rates in the DCN after tinnitus-inducing noise, implicates this structure as a site of phantom sound, or “tinnitus”, generation in animal models of tinnitus [4], [45], [46]. Because DCN neurons are more responsive to somatosensory stimulation following hearing damage [47], bimodal plasticity in DCN may play a role in somatic tinnitus, the modulation of the pitch and loudness of a phantom sound perception by pressure or manipulation of the head and neck [48], [49], [50]. In fact, the effect of bimodal stimulation, with Sp5 preceding tone stimulation, shifts from suppression in normal animals to enhancement in guinea pigs with behavioral evidence of tinnitus [4], suggests that bimodal plasticity may contribute to DCN hyperactivity in tinnitus. Although auditory nerve inputs to fusiform cells provide weaker drive after noise over-exposure, granule cell input to fusiform cells does not weaken, despite decreases in the input resistance of granule cells [51], perhaps due to cross-modal compensation [52], [53].

## Experimental Procedures

### Animals

Male pigmented guinea pigs (n = 5) from the University of Michigan colony (300–400 g; Ann Arbor, MI) were used in this study. All procedures were performed in accordance with the National Institutes of Health (NIH) *Guidelines for the Use and Care of Laboratory Animals* (NIH publication No. 80–23) and were approved by the University Committee on Use and Care of Animals at the University of Michigan.

### Surgical Approach and Electrode Placement

Guinea pigs were anesthetized (subcutaneous injection of ketamine and xylazine, 40 mg/kg, 10 mg/kg; at the incision site a subcutaneous injection of lidocaine, 4 mg/kg) and ophthalmic ointment applied to their eyes. Their heads were fixed in a stereotaxic frame using a bite bar and hollow ear bars were placed into the ear canals. Core temperature was maintained at 38°C. A left craniotomy was performed and a small amount of cerebellum was aspirated (leaving paraflocculus intact) to allow for visual placement of the recording electrode. Supplemental doses of ketamine and xylazine (I.M.) were administered at least hourly when indicated by response to a toe pinch. The guinea pig’s condition was monitored by assessment of body temperature, respiration and heart rates, and unit thresholds. After the completion of neural recording, the guinea pig was sacrificed by I.P. injection of sodium pentobarbitol followed by decapitation.

A concentric bipolar stimulating electrode (FHC, Bowdoin, ME) was dipped in fluorogold and placed stereotaxically into Sp5; −10 degrees below horizontal, 0.28+/−0.03 cm lateral from midline; 0.25+/−0.02 cm caudal from transverse sinus; 0.9+/−0.1 cm below surface of cerebellum. The location of the electrode was reconstructed post-mortem. A four-shank, thirty two-channel silicon-substrate electrode (site spacing = 100 um, shank pitch = 250 um, site area = 177 um^2^, impedance = 1–3 mOhms, NeuroNexus, Ann Arbor, MI) was placed at the DCN surface with each medial-to-lateral shank positioned within a different iso-frequency layer. The electrode was then lowered 0.8–1.0 um into DCN until the uppermost site on each shank responded to sound. In one guinea pig, after completing the recording protocol the DCN electrode was moved to a more medial location and a new frequency was selected for stimulation while the Sp5 stimulating electrode remained in place.

### Auditory and Somatosensory Stimulation

Neural activity in response to ***unimodal*** ** ***tones*** was recorded before and at 5, 15, and 25 minutes after the ***bimodal stimulation protocol*** (Fig. 1B). ***Tone*** signals (50 ms duration) gated with a cosine window (2 ms rise/fall time) were generated using Open Ex and an RX8 DSP (TDT, Alachula, FL) with 12 bit precision and sampling frequency set at 100 kHz. Sound was delivered to the left ear through the hollow ear bar by a shielded speaker (DT770, Beyer) driven by an HB7 amplifier (TDT, Alachula, FL). The system response was measured using a condenser microphone attached to the hollow earbar by a ¼” long tube approximating the ear canal. Sound levels were adjusted to account for the system response using a programmable attenuator (PA5, TDT, Alachula, FL) to deliver calibrated levels (dB SPL) at frequencies from 200 Hz to 24 kHz.

The ***bimodal stimulation protocol*** consisted of 500 trials of the 50 ms tones combined with electrical activation of Sp5 locations known to project to DCN [47]. Five biphasic (100 us/phase) current pulses at 1000 Hz were delivered to Sp5 through a concentric bipolar electrode using a custom isolated constant current source. The current amplitude was set to the highest level (range: 50–70 µA) that did not elicit movement artifact. The tone level (60–65 dB SPL) and frequency were fixed for the duration of the recording and were selected to reliably elicit responses to sound from most recording sites. The *bimodal interval* was defined as the onset of the Sp5 stimulus minus the onset of the tone, with negative values indicating Sp5-leading tone stimulation and positive values indicating tone-leading Sp5 stimulation. Varied bimodal intervals were used to assess stimulus-timing dependence of bimodal plasticity. During each recording session, the bimodal interval was randomly selected from the following intervals until all conditions were tested: −40, −20, −10, 0, +10, +20, +40, or +60 ms. For the unimodal control protocols, either the current amplitude was set to 0 uA or the sound level was set to 0 dB SPL.

### Spike Detection and Sorting

Voltages recorded from the multi-channel recording electrode were digitized by a PZ2 preamp (Fs = 12 kHz, TDT, Alachua, Fl, USA) and band-pass filtered (300 Hz –3 kHz) before online spike detection using a fixed voltage threshold set at 2.5 standard deviations above background noise (RZ2, TDT, Alachua, Fl, USA). Spike waveform snippets and timestamps were saved to a PC using Open Explorer (TDT, Alachua, Fl, USA). Waveform snippets were sorted using principal components of the waveform shape and K-means cluster analysis with fixed variance (95%) and 5 clusters (OpenSorter, TDT, Alachua, FL, USA). Clusters with a J2 value [4] above 1e−5 were not considered well isolated and were combined. Single units were identified by consistency of waveform shape and amplitude. Spikes up to 15 ms after the onset of the current stimulation were contaminated by electrical artifacts and ringing and excluded from all analyses. While multi-unit clusters could not be identified as isolated single units, the waveform shapes, amplitudes, and response properties were consistent over the duration of the recording.

### Experimental Design

To characterize unit responses to sound according to standard classification schemes [19], tone stimuli were presented before any Sp5 stimulation. Tone levels (0–85 dB SPL; 5 dB steps) and frequencies were varied (200 Hz –23 kHz; 0.1 octave steps) between trials (200 ms trial; 50 ms tone) with each condition repeated 10–20 times. The current amplitude for Sp5 stimulation was set at the highest amplitude that did not elicit ipsilateral facial twitches (60–80 µA). At the current amplitude presented, few units showed supra-threshold responses to somatosensory stimulation, but clearly subthreshold responses were elicited, as evidenced by the bimodal effects.

Unimodal trials were recorded at four time points: before, and 5, 15, and 25 minutes after the bimodal stimulation protocol (Fig. 1B). Responses were recorded to ***unimodal tones*** presented at the same level (60–65 dB SPL) as in the bimodal stimulation protocol (200 trials, 5 trials per second). Two minutes of ***spontaneous activity*** was also recorded at each time point before and after the bimodal stimulation protocol. All unimodal tones and rate level functions were at the same frequency used for bimodal stimulation. The entire recording block in Figure 1B lasted for 30–35 minutes with unimodal recordings at each time point lasting for 5–7 minutes and the bimodal stimulation protocol lasting for 4–5 minutes.

The recording block in Figure 1B was repeated randomly for each bimodal interval tested (−40, −20, −10, 0, 10, 20, 40, or 60 ms). In one guinea pig, control recording blocks were repeated in which unimodal tone or Sp5 stimuli replaced the bimodal stimuli. After the final recording block, the responses to unimodal tones were measured every 15–30 minutes for as long as possible to assess recovery after bimodal stimulation.

### Unit Characterization

All units were characterized by best frequency, threshold, frequency response map and temporal response patterns at best frequency [19]. Response maps were constructed by computing the sound-evoked firing rate during the 50 ms tone minus spontaneous firing rate measured during the last 50 ms of each trial. Excitation or inhibition was considered significant when the firing rate was greater than 2.5 standard deviations above or below the mean spike rate of all trials with no sound. Post-stimulus time histograms were constructed for each unit from 50–200 trials with the tone level 10–30 dB above threshold and frequency within 0.1 octave of the identified best frequency. Unit classification by receptive field and post-stimulus time histogram provide indirect evidence for the synaptic drive and intrinsic processing, respectively, of individual neurons in DCN.

### Statistical Analysis

A paired 1-tailed Student’s t-test was used to test the hypothesis that bimodal stimulation had a greater enhancing or suppressing influence than either unimodal tone or unimodal Sp5 stimulation. Linear regression analysis was used to fit changes in sound-evoked firing rates to a least-squares fit model of changes in spontaneous firing rates.

## References

[1] Shore SE (2005) Multisensory integration in the dorsal cochlear nucleus: unit responses to acoustic and trigeminal ganglion stimulation. Eur J Neurosci 21: 3334–3348. 16026471 10.1111/j.1460-9568.2005.04142.x

[2] Koehler SD, Pradhan S, Manis PB, Shore SE (2011) Somatosensory inputs modify auditory spike timing in dorsal cochlear nucleus principal cells. Eur J Neurosci 33: 409–420. 21198989 10.1111/j.1460-9568.2010.07547.x PMC3059071

[3] Kanold PO, Davis KA, Young ED (2011) Somatosensory context alters auditory responses in the cochlear nucleus. Journal of neurophysiology 105: 1063–1070. 21178001 10.1152/jn.00807.2010 PMC3295206

[4] Dehmel S, Pradhan S, Koehler S, Bledsoe S, Shore S (2012) Noise overexposure alters long-term somatosensory-auditory processing in the dorsal cochlear nucleus–possible basis for tinnitus-related hyperactivity? The Journal of neuroscience: the official journal of the Society for Neuroscience 32: 1660–1671. 22302808 10.1523/JNEUROSCI.4608-11.2012 PMC3567464

[5] Tzounopoulos T, Kim Y, Oertel D, Trussell LO (2004) Cell-specific, spike timing-dependent plasticities in the dorsal cochlear nucleus. Nat Neurosci 7: 719–725. 15208632 10.1038/nn1272

[6] Zhou J, Shore S (2004) Projections from the trigeminal nuclear complex to the cochlear nuclei: a retrograde and anterograde tracing study in the guinea pig. J Neurosci Res 78: 901–907. 15495211 10.1002/jnr.20343

[7] Haenggeli CA, Pongstaporn T, Doucet JR, Ryugo DK (2005) Projections from the spinal trigeminal nucleus to the cochlear nucleus in the rat. The Journal of comparative neurology 484: 191–205. 15736230 10.1002/cne.20466

[8] Tzounopoulos T, Rubio ME, Keen JE, Trussell LO (2007) Coactivation of pre- and postsynaptic signaling mechanisms determines cell-specific spike-timing-dependent plasticity. Neuron 54: 291–301. 17442249 10.1016/j.neuron.2007.03.026 PMC2151977

[9] Markram H, Gerstner W, Sjostrom PJ (2011) A history of spike-timing-dependent plasticity. Frontiers in synaptic neuroscience 3: 4. 22007168 10.3389/fnsyn.2011.00004 PMC3187646

[10] Roberts PD, Portfors CV, Sawtell N, Felix Ii R (2006) Model of auditory prediction in the dorsal cochlear nucleus via spike-timing dependent plasticity. Neurocomputing 69: 1191–1194.

[11] Bell CC, Han VZ, Sugawara Y, Grant K (1997) Synaptic plasticity in a cerebellum-like structure depends on temporal order. Nature 387: 278–281. 9153391 10.1038/387278a0

[12] Roberts PD, Leen TK (2010) Anti-hebbian spike-timing-dependent plasticity and adaptive sensory processing. Frontiers in computational neuroscience 4: 156. 21228915 10.3389/fncom.2010.00156 PMC3018773

[13] Young ED, Nelken I, Conley RA (1995) Somatosensory effects on neurons in dorsal cochlear nucleus. J Neurophysiol 73: 743–765. 7760132 10.1152/jn.1995.73.2.743

[14] Shore SE, Moore JK (1998) Sources of input to the cochlear granule cell region in the guinea pig. Hearing research 116: 33–42. 9508026 10.1016/s0378-5955(97)00207-4

[15] Weedman DL, Ryugo DK (1996) Projections from auditory cortex to the cochlear nucleus in rats: synapses on granule cell dendrites. The Journal of comparative neurology 371: 311–324. 8835735 10.1002/(SICI)1096-9861(19960722)371:2<311::AID-CNE10>3.0.CO;2-V

[16] Zhou J, Nannapaneni N, Shore S (2007) Vessicular glutamate transporters 1 and 2 are differentially associated with auditory nerve and spinal trigeminal inputs to the cochlear nucleus. J Comp Neurol 500: 777–787. 17154258 10.1002/cne.21208

[17] Caporale N, Dan Y (2008) Spike timing-dependent plasticity: a Hebbian learning rule. Annual review of neuroscience 31: 25–46. 10.1146/annurev.neuro.31.060407.125639 18275283

[18] Stein BE, Stanford TR, Ramachandran R, Perrault TJ Jr, Rowland BA (2009) Challenges in quantifying multisensory integration: alternative criteria, models, and inverse effectiveness. Experimental brain research Experimentelle Hirnforschung Experimentation cerebrale 198: 113–126. 19551377 10.1007/s00221-009-1880-8 PMC3056521

[19] Stabler SE, Palmer AR, Winter IM (1996) Temporal and mean rate discharge patterns of single units in the dorsal cochlear nucleus of the anesthetized guinea pig. Journal of neurophysiology 76: 1667–1688. 8890284 10.1152/jn.1996.76.3.1667

[20] Ding J, Benson TE, Voigt HF (1999) Acoustic and current-pulse responses of identified neurons in the dorsal cochlear nucleus of unanesthetized, decerebrate gerbils. Journal of neurophysiology 82: 3434–3457. 10601474 10.1152/jn.1999.82.6.3434

[21] Dahmen JC, Hartley DE, King AJ (2008) Stimulus-timing-dependent plasticity of cortical frequency representation. The Journal of neuroscience: the official journal of the Society for Neuroscience 28: 13629–13639. 19074036 10.1523/JNEUROSCI.4429-08.2008 PMC2663791

[22] Yao H, Dan Y (2001) Stimulus timing-dependent plasticity in cortical processing of orientation. Neuron 32: 315–323. 11684000 10.1016/s0896-6273(01)00460-3

[23] Sawtell NB (2010) Multimodal integration in granule cells as a basis for associative plasticity and sensory prediction in a cerebellum-like circuit. Neuron 66: 573–584. 20510861 10.1016/j.neuron.2010.04.018

[24] Fujino K, Oertel D (2003) Bidirectional synaptic plasticity in the cerebellum-like mammalian dorsal cochlear nucleus. Proc Natl Acad Sci U S A 100: 265–270. 12486245 10.1073/pnas.0135345100 PMC140946

[25] Sedlacek M, Tipton PW, Brenowitz SD (2011) Sustained firing of cartwheel cells in the dorsal cochlear nucleus evokes endocannabinoid release and retrograde suppression of parallel fiber synapses. J Neurosci 31: 15807–15817. 22049424 10.1523/JNEUROSCI.4088-11.2011 PMC3234102

[26] Manis PB (1989) Responses to parallel fiber stimulation in the guinea pig dorsal cochlear nucleus in vitro. J Neurophysiol 61: 149–161. 2918341 10.1152/jn.1989.61.1.149

[27] Manis PB (1990) Membrane properties and discharge characteristics of guinea pig dorsal cochlear nucleus neurons studied in vitro. J Neurosci 10: 2338–2351. 2376777 10.1523/JNEUROSCI.10-07-02338.1990 PMC6570364

[28] Doiron B, Zhao Y, Tzounopoulos T (2011) Combined LTP and LTD of modulatory inputs controls neuronal processing of primary sensory inputs. J Neurosci 31: 10579–10592. 21775602 10.1523/JNEUROSCI.1592-11.2011 PMC3164854

[29] Rhode WS, Smith PH, Oertel D (1983) Physiological response properties of cells labeled intracellularly with horseradish peroxidase in cat dorsal cochlear nucleus. The Journal of comparative neurology 213: 426–447. 6300199 10.1002/cne.902130407

[30] Smith PH, Rhode WS (1985) Electron microscopic features of physiologically characterized, HRP-labeled fusiform cells in the cat dorsal cochlear nucleus. The Journal of comparative neurology 237: 127–143. 4044890 10.1002/cne.902370110

[31] Kipke DR, Clopton BM, Anderson DJ (1991) Shared-stimulus driving and connectivity in groups of neurons in the dorsal cochlear nucleus. Hearing research 55: 24–38. 1752791 10.1016/0378-5955(91)90088-q

[32] Sherriff FE, Henderson Z (1994) Cholinergic neurons in the ventral trapezoid nucleus project to the cochlear nuclei in the rat. Neuroscience 58: 627–633. 7513389 10.1016/0306-4522(94)90086-8

[33] Shore SE, Helfert RH, Bledsoe SC Jr, Altschuler RA, Godfrey DA (1991) Descending projections to the dorsal and ventral divisions of the cochlear nucleus in guinea pig. Hearing research 52: 255–268. 1648060 10.1016/0378-5955(91)90205-n

[34] Mellott JG, Motts SD, Schofield BR (2011) Multiple origins of cholinergic innervation of the cochlear nucleus. Neuroscience 180: 138–147. 21320579 10.1016/j.neuroscience.2011.02.010 PMC3070814

[35] Zhao Y, Tzounopoulos T (2011) Physiological activation of cholinergic inputs controls associative synaptic plasticity via modulation of endocannabinoid signaling. The Journal of neuroscience: the official journal of the Society for Neuroscience 31: 3158–3168. 21368027 10.1523/JNEUROSCI.5303-10.2011 PMC3111389

[36] Kanold PO, Manis PB (1999) Transient potassium currents regulate the discharge patterns of dorsal cochlear nucleus pyramidal cells. J Neurosci 19: 2195–2208. 10066273 10.1523/JNEUROSCI.19-06-02195.1999 PMC6782577

[37] Dan Y, Poo MM (2006) Spike timing-dependent plasticity: from synapse to perception. Physiol Rev 86: 1033–1048. 16816145 10.1152/physrev.00030.2005

[38] Rhode WS (1999) Vertical cell responses to sound in cat dorsal cochlear nucleus. Journal of neurophysiology 82: 1019–1032. 10444694 10.1152/jn.1999.82.2.1019

[39] Bell C, Bodznick D, Montgomery J, Bastian J (1997) The generation and subtraction of sensory expectations within cerebellum-like structures. Brain Behav Evol 50 Suppl 117–31. 9217991 10.1159/000113352

[40] Requarth T, Sawtell NB (2011) Neural mechanisms for filtering self-generated sensory signals in cerebellum-like circuits. Current opinion in neurobiology 21: 602–608. 21704507 10.1016/j.conb.2011.05.031

[41] Roberts PD, Portfors CV (2008) Design principles of sensory processing in cerebellum-like structures. Early stage processing of electrosensory and auditory objects. Biol Cybern 98: 491–507. 18491162 10.1007/s00422-008-0217-1

[42] Oertel D, Young ED (2004) What’s a cerebellar circuit doing in the auditory system? Trends Neurosci 27: 104–110. 15102490 10.1016/j.tins.2003.12.001

[43] May BJ (2000) Role of the dorsal cochlear nucleus in the sound localization behavior of cats. Hear Res 148: 74–87. 10978826 10.1016/s0378-5955(00)00142-8

[44] Kanold PO, Young ED (2001) Proprioceptive information from the pinna provides somatosensory input to cat dorsal cochlear nucleus. J Neurosci 21: 7848–7858. 11567076 10.1523/JNEUROSCI.21-19-07848.2001 PMC6762891

[45] Kaltenbach JA, Zacharek MA, Zhang J, Frederick S (2004) Activity in the dorsal cochlear nucleus of hamsters previously tested for tinnitus following intense tone exposure. Neurosci Lett 355: 121–125. 14729250 10.1016/j.neulet.2003.10.038

[46] Brozoski TJ, Bauer CA, Caspary DM (2002) Elevated fusiform cell activity in the dorsal cochlear nucleus of chinchillas with psychophysical evidence of tinnitus. J Neurosci 22: 2383–2390. 11896177 10.1523/JNEUROSCI.22-06-02383.2002 PMC6758251

[47] Shore SE, Koehler S, Oldakowski M, Hughes LF, Syed S (2008) Dorsal cochlear nucleus responses to somatosensory stimulation are enhanced after noise-induced hearing loss. Eur J Neurosci 27: 155–168. 18184319 10.1111/j.1460-9568.2007.05983.x PMC2614620

[48] Sanchez TG, da Silva Lima A, Brandao AL, Lorenzi MC, Bento RF (2007) Somatic modulation of tinnitus: test reliability and results after repetitive muscle contraction training. Ann Otol Rhinol Laryngol 116: 30–35. 17305275 10.1177/000348940711600106

[49] Levine RA (1999) Somatic (craniocervical) tinnitus and the dorsal cochlear nucleus hypothesis. Am J Otolaryngol 20: 351–362. 10609479 10.1016/s0196-0709(99)90074-1

[50] Levine RA, Nam EC, Melcher J (2008) Somatosensory pulsatile tinnitus syndrome: somatic testing identifies a pulsatile tinnitus subtype that implicates the somatosensory system. Trends Amplif 12: 242–253. 18632767 10.1177/1084713808321185 PMC4134893

[51] Pilati N, Ison MJ, Barker M, Mulheran M, Large CH, et al (2012) Mechanisms contributing to central excitability changes during hearing loss. Proceedings of the National Academy of Sciences of the United States of America 109: 8292–8297. 22566618 10.1073/pnas.1116981109 PMC3361412

[52] Zeng C, Nannapaneni N, Zhou J, Hughes LF, Shore S (2009) Cochlear damage changes the distribution of vesicular glutamate transporters associated with auditory and nonauditory inputs to the cochlear nucleus. The Journal of neuroscience: the official journal of the Society for Neuroscience 29: 4210–4217. 19339615 10.1523/JNEUROSCI.0208-09.2009 PMC4487620

[53] Zeng C, Yang Z, Shreve L, Bledsoe S, Shore S (2012) Somatosensory projections to cochlear nucleus are upregulated after unilateral deafness. The Journal of neuroscience: the official journal of the Society for Neuroscience 32: 15791–15801. 23136418 10.1523/JNEUROSCI.2598-12.2012 PMC3501653

